# A human tau seeded neuronal cell model recapitulates molecular responses associated with Alzheimer’s disease

**DOI:** 10.1038/s41598-022-06411-4

**Published:** 2022-02-17

**Authors:** Elena Ficulle, Sarubini Kananathan, David Airey, Severine I. Gharbi, Neil Humphryes-Kirilov, James Scherschel, Charlotte Dunbar, Brian J. Eastwood, Emma Laing, David A. Collier, Suchira Bose

**Affiliations:** 1 Eli Lilly & Company, Erl Wood Manor, Sunninghill Road, Windlesham, GU20 6PH Surrey UK; 2 Eli Lilly & Company, Indianapolis, IN USA; 3 Eli Lilly & Company, Alcobendas, Spain; 4 Present Address: Eli Lilly & Company, 8 Arlington Square West, Bracknell, RG12 1PU UK; 5 Present Address: UK Dementia Research Institute, UCL, London, WC1E 6BT UK; 6 Present Address: C4X Discovery Ltd, London, NW1 1AD UK; 7 Present Address: GSK, Gunnels Wood Rd, Stevenage, SG1 2NY UK

**Keywords:** Molecular biology, Protein folding, Protein aggregation, Neurological disorders

## Abstract

Cellular models recapitulating features of tauopathies are useful tools to investigate the causes and consequences of tau aggregation and the identification of novel treatments. We seeded rat primary cortical neurons with tau isolated from Alzheimer’s disease brains to induce a time-dependent increase in endogenous tau inclusions. Transcriptomics of seeded and control cells identified 1075 differentially expressed genes (including 26 altered at two time points). These were enriched for lipid/steroid metabolism and neuronal/glial cell development genes. 50 genes were correlated with tau inclusion formation at both transcriptomic and proteomic levels, including several microtubule and cytoskeleton-related proteins such as Tubb2a, Tubb4a, Nefl and Snca. Several genes (such as Fyn kinase and PTBP1, a tau exon 10 repressor) interact directly with or regulate tau. We conclude that this neuronal model may be a suitable platform for high-throughput screens for target or hit compound identification and validation.

## Introduction

Alzheimer’s disease (AD) is the most common cause of dementia with key neuropathological features that include senile plaques comprised of amyloid β (Aβ) peptides and intracellular aggregates of the soluble microtubule-associated protein tau, which misfolds into insoluble, filamentous and hyperphosphorylated inclusions (neurofibrillary tangles or NFTs)^1^. Aβ pathology forms 10–20 years prior to symptom onset, while the distribution and spread of tau pathology correlates with cognitive decline in AD^2,3^. In AD, NFTs first accumulate in the locus coeruleus, from where they appear to spread to the entorhinal cortex, hippocampus and neocortex. This differential distribution underlies the Braak stages of tau pathology^4,5^. According to this staging system, the initial accumulation of hyperphosphorylated tau occurs before the disease becomes symptomatic; the propagation of tau pathology beyond the entorhinal cortex and locus coeruleus by neuron-to-neuron transmission is initiated after accumulation of a high Aβ load in iso-cortical regions and is associated with symptoms of AD, according to pathological, clinical and biomarker data^6^.

Tau NFTs occur in several human neurodegenerative diseases collectively known as tauopathies. These include AD, argyrophilic grain disease (AGD), progressive supranuclear palsy (PSP), corticobasal degeneration (CBD), tangle-only dementia (TD), Pick’s disease (PiD), and chronic traumatic encephalopathy (CTE)^7^. Mutations in *MAPT,* the tau gene*,* cause familial forms of frontotemporal dementia, confirming that tau protein dysfunction is sufficient to cause neurodegeneration and dementia^8,9^. *MAPT* mutations cause the formation of inclusions made of full-length hyperphosphorylated filamentous tau; depending on the nature of the *MAPT* mutation, the inclusions contain all six tau isoforms, or predominantly 4R tau^10^.

Cellular and animal models recapitulating features of tauopathies provide a useful tool to investigate the causes and consequences of tau aggregation, understanding disease mechanisms, and screening and profiling compounds that interfere with tau inclusion formation. Neuronal models that recapitulate tau inclusion formation in cells that do not overexpress the tau protein, are particularly suited to studying mechanisms of tau-induced pathology and toxicity as a model for tauopathies. Such models have been described recently in the literature. Guo et al. described the use of primary mouse cortical cultures where they induced tau inclusion formation using human AD-derived tau seeds^11^. More recently, we and others have shown that primary rat cortical neurons can also form tau inclusion formation upon induction with human AD-derived tau seeds^12,13^. Here we have used this neuronal model to investigate changes in gene and protein expression over time to identify biological processes and/or pathways that could be modulated as potential therapeutic intervention for AD and other disorders of tau aggregation.

## Methods

### Preparation of hAD seeds

Post-mortem human brain tissues from AD patients were obtained from Manchester Brain Bank and King’s College London Neurodegenerative Diseases Brain Bank. Consent was obtained from legal guardian(s) for the involvement of Alzheimer’s disease patients (vulnerable population). The brain banks were responsible for obtaining informed consent from legal guardian(s). The Manchester Brain Bank Management Committee (University of Manchester) and the MRC London Neurodegenerative Disease Brain Bank (King’s College London) assessed the applications on their scientific merit and ethical use of tissues and granted approval for the specified use. All experiments were performed according to HTA (Human Tissue Authority) guidelines. Both Brain Banks have generic ethics committee approval to function as research tissue banks, which means that they can provide stem cells and tissue samples to UK-based researchers for a broad range of studies without the need for the researchers to obtain their own ethics approval. Human AD seeds were prepared from post-mortem brain tissue using a protocol adapted from Greenberg and Davies^13^. Post-mortem human brain tissue was obtained as described above after confirmation of consent and histological confirmation of tau pathology. The pool of cortical tissues used were obtained from ∼ 20 patients with Alzheimer's disease, modified Braak (Brain Net Europe) stage 6 with moderate amyloid angiography. The average age was 71 years, and the gender was mixed male and female. The samples chosen for the preparation were those with the highest levels of AT8-positive Tau (0.5 μg/ml) as determined by AlphaScreen. All human tissue was handled in compliance with the regulations set by the HTA Human Tissue Act 2004.

### Rat cortical neuron treatment with human AD tau seed and protein/RNA extraction

Rat cortical neurons (RCNs) were cultured from E18 Sprague Dawley rats and plated in 2 mL of neurobasal media (Invitrogen) at 1.7 × 10^6^ cells/well in 6-well plates (#354413, Corning). Four plates per time point were harvested at Days In Vitro (DIV) 3, 7 and 14; two for RNA and two for protein extraction. In parallel, RCNs were plated in 100 μL of neurobasal media (Invitrogen) at 40 K cells/well on four poly-d-lysine-coated (PDL) 96-well plates (#354640, Corning) for high content imaging, one per time point. At DIV 3, cells were treated with 18 nM of human AD (hAD) seed (preparation adapted from Greenberg and Davies^14^). The seed was first sonicated for 1 min at 20% amplitude, with 1-s pulses (QSonica Probe) and then filter sterilized with a 0.22 μm filter and syringe (PN4602, Pal Acrodisc). After 5 h, the 6-well DIV 3 plates were washed twice with Dulbecco's phosphate-buffered saline (DPBS) and RNA was extracted using the Ambion RNAqueous Total RNA isolation kit (Thermofisher, #AM1914). RNA concentration was determined with a NanoDrop spectrophotometer (Thermo Fisher) and quality was assessed with an Agilent Bioanalyser (Agilent, RNA 600nano, 5067-511). For proteomic analysis the total lysate was centrifuged for 10 min at 13,000 rpm and the supernatant was used for proteomic analysis. Proteins were extracted using RIPA buffer (Sigma, #R0278) supplemented with 0.5 mM Phenylmethylsulphonyl fluoride (PMSF) protease inhibitor, 2 mM Sodium ortho-vanadate, Phosphatase Inhibitor Cocktail 1 (Sigma, #P2850), Phosphatase Inhibitor Cocktail 2 (Sigma, #P5726) and 1 tablet of Complete EDTA free Protease Inhibitor Cocktail (Roche, #11697498001)). At the same time, the 96-well plate was washed and fixed with 100% ice-cold methanol for 15 min. At DIV 7 and 14, the appropriate plates were extracted and fixed as described above. All animal procedures were performed in accordance with the Animals (Scientific Procedures) Act 1986 and were reviewed by the internal Animal Welfare and Ethical Review Body (the Eli Lilly Animal Welfare Board) to ensure they comply with ethical and welfare standards. Procedures were in compliance with the ARRIVE guidelines.

### Immunofluorescence and high content imaging

RCNs were blocked with 100 µL/well of Intercept Blocking Buffer (LI-COR) containing 0.1% Triton for 1 h at room temperature. The primary tau antibody T49 (#MABN827, Millipore) was prepared at a 1:1000 and incubated overnight at 4 °C with gentle agitation. Cells were then washed and incubated for 1 h at room temperature with the secondary antibody, Goat anti-mouse IgG1 647 Alexa fluor-conjugated antibody, as well as Hoechst 33342 (both Invitrogen), both prepared at 1:1000. Cells were washed with DPBS and imaged with the Operetta CLS High-Content Analysis System (Perkin Elmer). Images were typically taken using the 20X water objective, with 9 fields of view per well, and a Z-stack of 5–7 slices. The images were analyzed using Harmony software (Perkin Elmer) and the final readouts were “T49 inclusion count normalized to the live nuclei count” and “% of live nuclei” (determined by a nuclei area threshold) normalized to the DIV 14 time point. Data were plotted in Prism 8 (GraphPad).

### RNA-seq analysis

RNA sequencing (paired-end, 100nt, 30–40 million generated reads, PolyA) was performed by Covance Genomics Laboratory (CGL; Redmond, WA 98052 USA). RIN numbers for the extracted RNA ranged from 7.7 to 10 (average 9.5). Raw RNA-seq reads were processed to obtain gene-level RNA-abundance values using a pipeline developed by Eli Lilly and Company. FASTQ files were checked for integrity and quality, based on base quality, base composition, heterologous organisms, rRNA/mitochondrial/viral and adaptor content, before aligning to the *Rattus norvegicus* reference genome assembly using GSNAP^15^. All samples passed additional, post-alignment quality control (3′ bias, flow cell bias, template length, sample relatedness, species). Quantification was performed at the exon level (using NCBI-based gene models) and Log_2_ median-exon counts used to denote gene-level RNA-abundance for 18,049 genes. Quantile normalization across all gene-level summarized samples was performed. Unsupervised clustering approaches (PCA, Hierarchical clustering based on Euclidean distance and Pearson correlation) did not identify any groupings associated with technical factors such as lane, RNA-quality etc.

A treatment by DIV factorial model was used for differential expression analysis. There were three independent samples per treatment*DIV combination. Plate effects were handled by a mixed model when detected (R lmer, R version 3.52 https://www.r-project.org/nosvn/pandoc/lme4.html), otherwise a linear model was used (R lm). Within gene family-wise error rate (FWER) for pairwise treatment contrasts at each DIV level (i.e., Seed-Control@3, 7, or 14 DIV) were controlled by a Tukey procedure (R emmeans). The minimum Tukey corrected p-value within a gene was then used to control the false discovery rate (FDR) to 5% across genes (R p.adjust (,method=”fdr”)). This produced a set of FDR adjusted p-values, one per gene. To resolve which of the multiple contrasts within a gene were significant, both in terms of FWER and FDR, the maximum of the minimum FWER p-values was found for FDR adjusted p-values < 0.05 and called p*. FWER p-values less than p* identified significant contrasts overall. Fold change effect sizes from model predicted means were computed to aid summarization of results.

### Proteomics analysis

Typically, 30–80 ug of cellular extract was used for proteomics profiling. Prior to protein digestion, samples were precipitated with methanol-chloroform following a protocol adapted from Wessel and Flügge^16^. Briefly, 100 µL of sample was mixed with 4 volumes of methanol, 1 volume of chloroform and 3 volumes of water, vortexed and centrifuged at 16,000×*g* for 2 min to separate the protein fraction. The aqueous top layer was discarded, and protein pellet washed in 4 volumes of methanol prior to collecting protein pellet following another round of centrifugation at 16,000×*g* for 2 min. The dried protein pellet was then resuspended in 8 M urea, 20 mM Hepes, pH 7.4. Once the pellet was homogenized, urea concentration was diluted to 2 M final concentration in triethylammonium bicarbonate buffer (TEAB), pH 8.5. Proteins were reduced with 10 mM dithiothreitol (DTT) and alkylated in 55 mM iodoacetamide (IAA) both in a 50 mM TEAB solution. Trypsin was added (1:100 enzyme to protein ratio; Trypsin MS Grade, Thermo Fisher Scientific) and incubated at 37 °C for 18 h. The reaction was terminated by adding 0.5% trifluoroacetic acid (TFA) and peptides were dried by speed-vacuum centrifugation. Tryptic peptides were cleaned up using OMIX C18 100 µL pipet-tip (Agilent Technologies) following vendor’s procedure. Eluted peptides were dried in speed-vacuum centrifugation and stored at − 20 °C prior to LC–MS/MS analysis.

Samples were analysed by LC–MS/MS using a nano HPLC chromatography system (RSLC nano UltiMate™ 3000, Thermo Fisher Scientific) coupled online to a Q Exactive-HF mass spectrometer (Thermo Fisher Scientific) using a Nanospray Flex ion source. Tryptic peptides were separated by reversed phase chromatography using a trapping column (Acclaim PepMap C18, Thermo Fisher Scientific) and analytical column (LC-Acclaim PepMap C18, 75 µm, Thermo Fisher Scientific). An estimated of 0.5 µg of peptide mixture was injected and nano-HPLC conditions were as follows: Loading pump was operated at 20 µL/min in 0.05% TFA in water, and nanopump at 300 nL/min flowrate during a total run duration of 270 min with linear gradient elution conditions of: 10–25% B in 225 min (A = 0.1% Formic acid (FA) in water, B = 0.1% FA in 80% acetonitrile).

Data was acquired on a top 20 data-dependent analysis mode with following acquisition parameters: MS survey scan range was acquired at 300–1300 m/z range with resolution of 60,000 at m/z 200, maximum ion injection time 100 ms, AGC target of 3 × 106. Ion fragmentation was done in higher energy collision dissociation (HCD) mode, MS/MS scan time was 100 ms with AGC target of 1 × 105 at a resolution of 15,000 at m/z 200 and isolation window of 1.3 m/z. Charge state was set to 2–5, dynamic exclusion 30 s and normalized collision energy (NCE) was 28.

MS/MS data were analysed using the Proteome Discoverer data analysis program, version 2.4 (Thermo Fisher Scientific) in a label free quantification mode. MS/MS files were searched against the Uniprot protein database from Rat (Rattus norvegicus 10116, reference proteome ID UP000002494 accessed on 12/03/2020) with enzyme specificity set for Trypsin, with target FDR criteria was 0.01 (strict) and 0.05 (relaxed). Cysteine carbamidomethylation was set as fixed modification and variable modifications were N-terminal protein acetylation, methionine oxidation and phosphorylation at serine, threonine, tyrosine (STY). Peptide Percolator was used for peptide validation based on the posterior error probability (PEP) score. As additional criteria, proteins were identified with a cut-off of at least two high quality peptides identified.

Protein quantification was based on peptide abundance measured from precursor ion peak intensity and then protein abundance was calculated from summed abundance of all identified peptides for a given protein. Data normalization was applied based on total peptide amount between all samples within the dataset. Data was exported as an Excel report file and further bioinformatics analysis carried out. Significantly differentially expressed genes were mapped to proteins using the Uniprot mapping tool^17^ to map gene Entrez IDs to protein accession IDs.

### qRT-PCR to validate RNA sequencing

Extracted RNA was normalized to the lowest concentration among the samples (700 ng) and reverse transcription (RT) was performed to convert RNA to cDNA. The master mix containing the RT components was prepared in advance (Table 1) and 30 μL were added to 70 μL of RNA diluted in water. The RT protocol run on the thermocycler includes 25 °C, 10 min; 37 °C, 60 min; 75 °C, 5 min; 4 °C hold. At the end of the run the cDNA plate was used for qRT-PCR.

**Table 1:** Master mix reagents for qRT-PCR.

Component	Source	Catalogue no.	Stock conc.	Final conc.
dNTP mix	Ambion	1502025	2.5 mM each	1 mM
Random hexamers	Invitrogen	100026484	50 µM	5 µM
10 × RT buffer	Life Technologies	Part of AM2044	10X	1X
M-MLV RT	Life Technologies	AM2044	100 U/μL	1.5 U/μL
RNase inhibitor	Life Technologies	N8080119	20 U/µL	0.4 U/μL

The qRT-PCR probes were multiplexed, and automation was used to prepare the two plates. Probes used were: Kif14, Idi1, Pchdb5, Sqle, Aebp1, A2m, Bcl6 and Gstk1 (Table 2).

**Table 2:** Probes used to validate RNA sequencing.

Gene	Taqman probe	Serial number, reporter dye
Kif14	Rn01490403_m1	4331182, FAM
Idi1	Rn00585526_m1	4331182, FAM
Pcdhb5	Rn02395723_s1	4351372, FAM
Sqle	Rn00567532_m1	4331182, FAM
Aebp1	Rn01443083_m1	4331182, FAM
A2m	Rn01640253_g1	4351372, FAM
Bcl6	Rn01404339_m1	4331182, FAM
Gstk1	Rn00710510_g1	4331182, FAM
Gapdh	Rn01775763_g1	4448490, VIC

To prepare the master mix used by the first robot (QIAgility) each RNA was mixed with the two probes (specific probe and Gapdh to normalize), Taqman MasterMix and water.

The QIAgility robot was used to plate the qRT-PCR reaction in the 96-well plate by adding 2 ng cDNA sample (4 μL) with 36 μL master mix for each primer. Controls containing water in place of either the cDNA or the reverse transcriptase enzyme were run alongside samples. The EpMotion robot was used to convert this into quadruplicate in a 384-well plate and the reaction was run on the QuantStudio5 Real-Time PCR system (Applied Biosystems). Data was analysed by the ΔΔCt method^18^, which manually normalises to each internal Gapdh Ct value as well as normalising each seeded condition to its own unseeded control. Results were then plotted in Prism 8 expressed as a Log_2_ fold change, calculated as the Log_2_ of the ratio between the seeded and unseeded control for each time point.

### Functional interpretation of transcriptomic data

Significantly differentially expressed genes were analysed for enrichment of GO Biological Processes (GOBP), as defined in Metabase^19^, using the genes quantified in the respective transcriptomic assessments as the background. Benjamini and Hochberg multiple-testing correction was applied and any GOBPs with an FDR-adjusted p-value < 0.05 were defined as significantly enriched.

Canonical Pathway enrichment and activity was assessed using Ingenuity Pathway Analysis software (Qiagen; version 51963813)^20,21^. Gene identifiers and expression values were used as the input dataset and the ‘Core Analysis’ function was run. Part of this analysis looks for significant enrichment of genes in canonical pathways manually curated from literature, using the right-tailed Fischer’s exact t-test to calculate significance with p < 0.05 as the threshold. These known pathways have edge direction information indicating the relationship between each pair of molecules within the pathway. This directional edge information is compared with measured data to predict the activity (increased or decreased) of each canonical pathway identified.

### Annotation of genes and proteins

To understand relevance at the gene level, we further annotated the 26 genes significantly differentially expressed at two time points in the transcriptomic analysis and 50 genes that were significantly changed in both the transcriptome and proteome at one or more time points (Supplementary Files 1, 4, 5). Genes were annotated with protein function using GO terms (accessed through UNIPROT^17^), as well as information on tissue and cellular expression; cell type expression correlates^22–24^; and association with genetic diseases (https://omim.org/; Supplementary File 5).

### URLs

For Uniprot, see https://www.uniprot.org/; for Cell Type Expression Correlates see http://oldhamlab.ctec.ucsf.edu/ ; for Brain RNA-Seq see https://www.brainrnaseq.org/; for GTEx Portal, see http://www.gtexportal.org/; for The Human Protein Atlas see https://www.proteinatlas.org/; for Human genetic disorders (OMIM) see https://omim.org/. For Parkinson’s disease genetics see https://pdgenetics.shinyapps.io/GWASBrowser/.

## Results

### Time dependent increase in rodent tau inclusion formation upon induction by hAD-derived tau seeds

**Figure 1:**
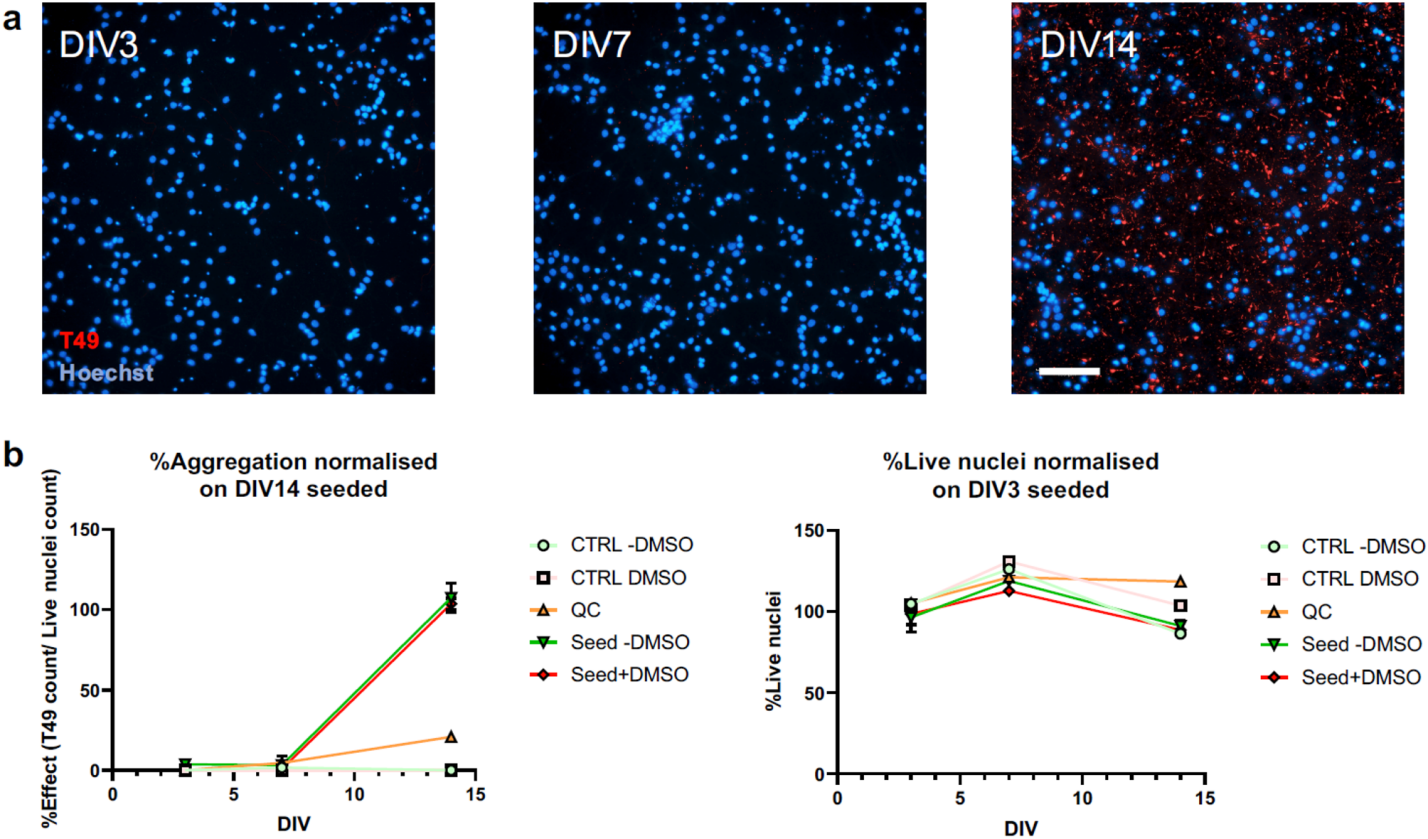
Human AD seeding time course in RCNs in a 96-well assay format. High-content imaging shows how neuritic, thread-like aggregates form over time that can be detected with the rodent-specific Tau antibody, T49 (**a**). The percentage of effect expressed as the ratio between T49 positive aggregates, and the count of live nuclei has been normalised on the amount of aggregates at DIV 14. Scale bar 100um (**b**).

Recently we showed that hAD-seeded RCNs can be used as a model to study endogenous tau aggregation and propagation^12^**.** Here we used that model to understand changes in gene and protein expression in response to aggregation induction with hAD-derived tau seeds at DIV 3, 7, and 14 to delineate early changes associated with tau pathology. RCNs were cultured and samples for transcriptomic and proteomic analysis taken at DIV 3, 7, and 14. In parallel, we used high-content imaging using the rodent specific antibody T49 (Fig. 1A) to assess tau aggregation. Consistent with previous findings^11,12^, we observed a time-dependent increase in neuritic, thread-like inclusions following induction with hAD-derived tau seeds that increased exponentially at the later time point (Fig. 1B). We also observed a decrease in the percent of live nuclei during the time course that was independent of hAD seed treatment and more likely to be a response of cells grown in culture for long periods of time.

### The effect of seed and tau aggregation on the transcriptome

**Figure 2:**
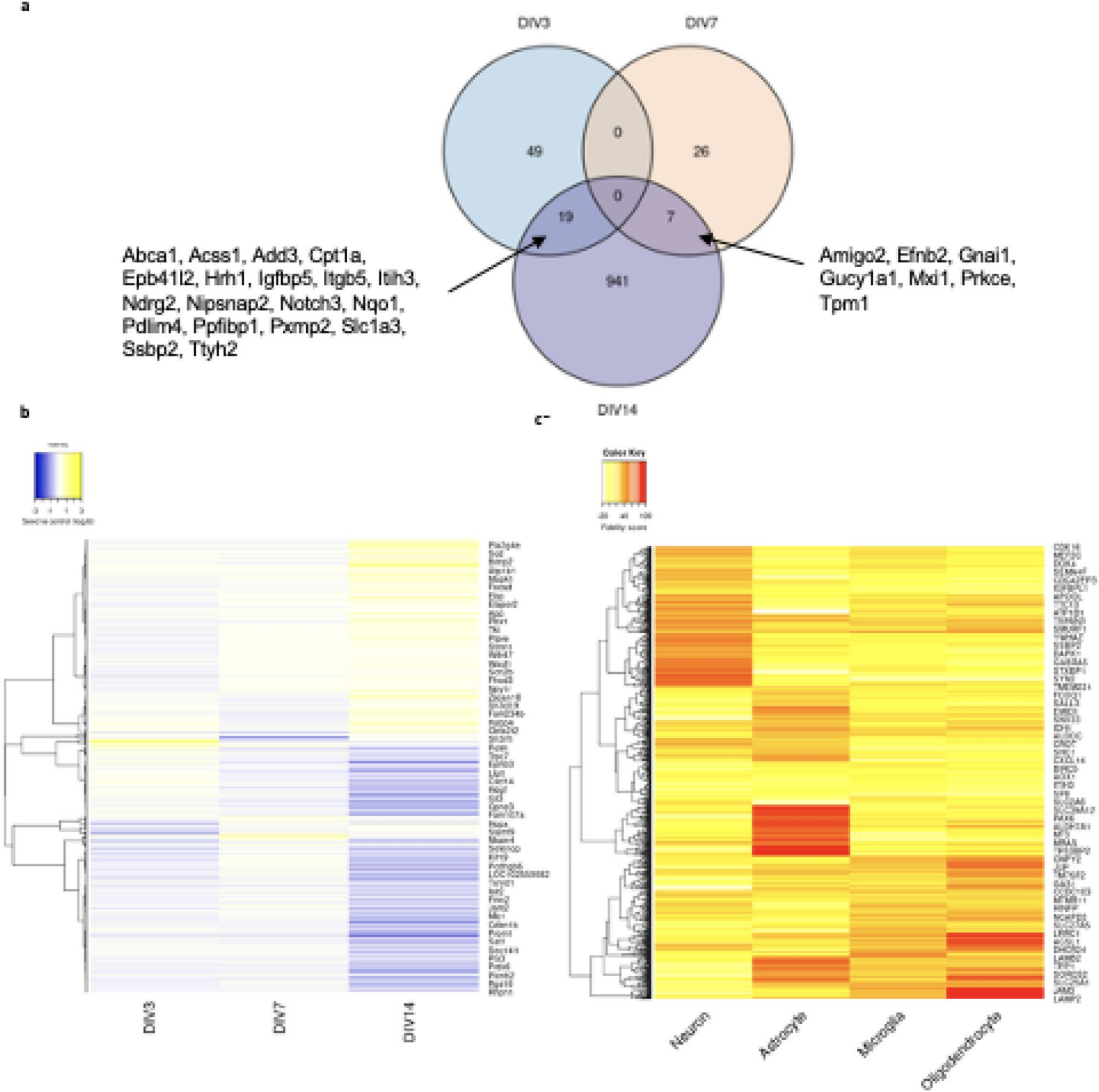
Analysis of the 1075 significantly differentially expressed genes from differential expression analysis. (**a**) Venn diagram showing the overlap of significant genes identified at DIV3, DIV7 and DIV14. (**b**) Heatmap of differentially expressed genes between seed and control at timepoints DIV 3, DIV 7 and DIV 14. Row clustering was applied based on Pearson correlation between genes. Underlying data to generate plot can be found in Supplementary File 2. (**c**) Heatmap of fidelity scores from Cell Type Correlates Oldham Lab^22–24^ for cell type specific expression of differentially expressed genes. Row clustering was applied based on Euclidean distance between genes.

In total, 1,075 genes were identified as significantly (p < 0.05) differentially expressed between seed and control in at least one of the sampled time points (Fig. 2, Supplementary Fig. 2, Supplementary File 2). Of the 1,075, none were shared between DIV 3 and DIV 7, nineteen (Ndrg2, Nqo1, Hrh1, Pdlim4, Add3, Igfbp5, Cpt1a, Itgb5, Ttyh2, Slc1a3, Pxmp2, Acss1, Epb41l2, Ppfibp1, Abca1, Ssbp2, Nipsnap2, Itih3, Notch3) were shared between DIV 3 and DIV 14, and seven (Tpm1, Gnai1, Mxi1, Prkce, Amigo2, Efnb2, Gucy1a1) were shared between DIV 7 and DIV 14 (Fig. 2a). Most of these significant changes (991 genes; ~ 92% of the 1075), were observed at DIV 14. Assessing expression profiles across DIV, 341 of the 1075 genes identified as significantly differentially expressed in at least one timepoint presented an increasingly up-regulated profile with increasing pathology (Spearman correlation rho between expression profile and pathology of 1 or − 1, respectively), while 253 genes presented an increasingly down-regulated profile. We subsequently used qRT-PCR to confirm the differential expression trend of 8 genes shown to have a continuous pattern of increasing or decreasing differential expression over time (with at least one timepoint significant; Table 2). The chosen genes were multiplexed with Gapdh as a housekeeping gene to normalize their expression and for each time point, the fold-changes of each gene were calculated. These results confirm and validate the RNA-seq analysis, as most of the genes show the same trends observed in the RNA-seq results. A2m, Aebp1, Bcl6 and Gstk1 show a decrease in fold-change over time, while Kif14, Idi1, Sqle and Pcdhb5 show an increase (Supplementary Fig. 1).

Submitting the entire set of significantly differentially expressed genes for GOBP functional enrichment analysis to Metabase, 134 biological processes were significantly (FDR corrected p-value < 0.05) enriched. Processes were associated with lipid, steroid and other metabolic processes, neuron and nervous system development and differentiation, and glial cell development and differentiation (Supplementary File 3). Canonical pathway analysis (Ingenuity Pathway Analysis, IPA) on RNA-seq data identified 267 canonical pathways as being significantly enriched in this dataset (p < 0.05; right-tailed Fisher’s exact t-test, Supplementary File 3). This analysis showed that of those 267 pathways, 114 have increased activity, 63 have decreased activity and 90 do not have enough information to show direction. The top 4 significantly activated pathways relate to cholesterol biosynthesis while inhibited pathways include protein kinase A and Il-22 signalling.

### The effect of seed and tau aggregation at the protein level

Next, for the genes that showed a significant response to treatment in at least one condition, we compared the fold-change at RNA and protein levels. Label-free proteomics analysis of DIV 3, 7 and 14 seeded and unseeded cells was performed. We identified a total of 3743 proteins of which 1941 were measured in all biological replicates (Supplementary File 1). Of the genes significantly differently expressed at the RNA level, 366 were quantified at the protein level (~ 34% of the 1075). Fifty genes exhibited increasing up-regulation or down-regulation over time with pathology at both the transcriptomic and proteomic level (Spearman Correlation rho between protein abundance and pathology of 1 or − 1 respectively).

## Discussion

Cellular assays that can measure uptake of tau or the formation of aggregates or inclusions are potentially powerful tools for the identification of therapeutic agents such as small molecules, as they will be amenable to compound screening approaches. Cell-based assays have advantages over cell-free approaches because they may more closely mimic the cellular environment in vivo. Several cellular assays have been developed for tau toxicity^25–28^ and some of these have been used to screen compounds against measures of tau pathology^13,29^ or perform CRISPR screens^30,31^. The most comprehensive of these studies used two cell-based assays of tau inclusion; HEK293 tau overexpression and a primary rat cortical neuron assay with physiological tau expression, to screen for compounds that modulate tau inclusion formation and provided support for the use of neuronal cells in such an assay^13^. However, few studies have attempted to look at the effects of tau seeding at the molecular level in cellular assay systems such as neurons.

Here we performed transcriptomic and proteomic analyses to assess the utility of a human tau seeded primary rat cortical neuron model as a platform with which to investigate molecular mechanisms associated with AD. Endogenous tau inclusion formation was induced using tau seeds derived from human AD patient brains and monitored over time. Consistent with our previous findings, we observed a time-dependent increase in endogenous tau inclusions in this model^12^. Differential gene expression analysis between seeded and unseeded cells at three time points (DIV 3, 7 and 14) was performed to understand time-dependent changes in gene expression associated with tau inclusion formation. Subsequently, we performed proteomic analysis to confirm whether changes in the transcriptome were also observed in the proteome.

A limitation of the study is that we used unseeded cells as our control rather than age-matched human brain tissue, which would have served as a better control. However, we have previously shown that tau aggregation is directly proportional to the level of pathological tau present in seeds^32,33^ as seeds generated from non-mutated wild-type mouse brains or human tau-depleted seeds did not result in significant intracellular aggregates.

Experiments were performed in embryonic rat primary neuronal cultures which are mixed cultures comprised of neuronal and glial cells, however the majority are neuronal. Therefore, the changes in gene and protein expression observed upon hAD seed treatment are not exclusive to neuronal cells and will include changes in glial cells (Fig. 2c).

Pathway enrichment and activity analyses of the 1075 genes significantly differentially expressed in one or more timepoints identified pathways and pathway responses that are in keeping with the literature. The top 4 significantly activated pathways relate to cholesterol biosynthesis, which is thought to be dysfunctional in AD^34,35^. The role of cholesterol in AD pathology is poorly understood; however, the strongest genetic risk factor for AD, APOE, is a key transporter of extracellular cholesterol in humans, and several other Alzheimer’s risk genes, including clusterin (Apolipoprotein J), ABCA7 and ABCA1, are involved in cholesterol transport and regulation. A recent metabolomic and transcriptomic study of brain cholesterol homeostasis in late-stage AD suggests reduced de novo cholesterol biosynthesis may occur in response to impaired enzymatic cholesterol catabolism and efflux, in order to maintain brain cholesterol levels in AD^36^.

Inhibited pathways include the cAMP-dependent protein kinase A (PKA) pathway, which has also been implicated in AD, especially in relation to tau. PKA is a pro-apoptotic kinase allosterically activated by cAMP which phosphorylate multiple proteins including cAMP response element binding protein (CREB), whose target genes are required for the synaptic plasticity mediating long-term memory formation. CREB-mediated gene expression is impaired in the brains of both AD mouse models and human AD brain^37^, consistent with our model. PKA can also phosphorylate tau, including within its microtubule-binding domains, at Ser-262 and Ser-356^38,39^. PKA phosphorylates a total of 16 sites on tau, ten of which are also present on PHF-tau^40^. In AD brains, PKA proteins are activated and co-localized in NFTs (reviewed in^41^), further implicating PKA in tangle formation. The inhibition of the PKA pathway is in the opposite direction of that seen in AD brain. This may represent an acute response at the cellular level to the accumulation of tau, which contrasts to the activation and accumulation of PKA in tangles seen in the aging brain.

This highlights the ability of this neuronal model to recapitulate relevant biological mechanisms, at least at the pathway level. To understand relevance at the gene level, we focused our attention on the 26 genes significantly differentially expressed at two time points and 50 genes that were significantly changed in both the transcriptome and proteome at one or more time points. Only one gene, Slc1a3, was significantly changed at two time points in the transcriptomic analysis and in the proteomic analysis. In each of these sets we were able to recover genes that have been associated with AD either genetically and/or functionally.

Of the 26, four are known to have genetic links to CNS disorders; ADD3 (cerebral palsy with spastic paraplegia), SLC1A3 (Episodic ataxia, type 6), NOTCH3 (Cerebral arteriopathy with subcortical infarcts and leukoencephalopathy 1) and ABCA1 (GWAS association with AD; https://omim.org/). NOTCH3 mutations have been associated with vascular dementia^42^ and several potentially pathogenic genetic variants of uncertain significance have been identified in early-onset AD, atypical dementia patients, or Lewy body disease^43,44^. Studies in AD models have implicated ABCA1 mechanistically in the disease^45^ and ABCA1 is a major protein in the ApoE pathway, which contains the major genetic risk gene for AD, *APOE4*. ApoE lipidation is controlled by the activity of ABCA1, and the APOE4 genotype is thought to impair the recycling of ABCA1 and therefore its ability to lipidate ApoE^46^. In addition, ABCA1 small molecule agonists or inducers have been proposed as treatments for AD^47,48^. Functionally, several of the 26 genes are associated in synapse processes, namely Ndrg2 (postsynapse organization), Nqo1 (positive regulation of neuron apoptotic process), Hrh1 (neurotransmitter receptor activity, regulation of synaptic plasticity), Pdlim4 (excitatory chemical synaptic transmission), Slc1a3 (positive regulation of synaptic transmission), Ppfibp1 (synapse organisation), Gnai1 (negative regulation of synaptic transmission), Amigo2 (negative regulation of neuron apoptotic process; negative regulation of programmed cell death; positive regulation of synapse assembly) and Efnb2 (negative regulation of neuron projection development; positive regulation of neuron death; presynapse assembly; Gene Ontology, selected terms).

50 genes showed changes at both the transcript and proteomic level, which could be an increase in both transcript and protein (22), a decrease in both (8), an increase in transcript and decrease in protein (18), or a decrease in transcript and increase in protein (2). These sets were too small for formal gene set analysis, so they were examined qualitatively to look for biological themes. Each of these genes was annotated with protein function using GO terms, accessed through Uniprot (24/01/2021 and 08/12/2021), as well as information on tissue and cellular expression^22^ human brain^49^ (cerebral cortex). 26 of these genes were enriched or enhanced in the brain (INA, LANCL1, MAPT, NEFL, NSF, SNCA, SPOCK1, STMN1, SYN1, SYN2, SYT1, GPM6B, PCDHGC3, SLC1A3, CAMK2B, CRMP1, DYNC1I1, NCAN, RAB6B, L1CAM, MAP4K4, PHF24, RAB33A, TUBB2A, TUBB4A, ATP1A3), which is to be expected since the data is focused on rat brain cortical neurons, although other brain cell types (microglia, oligodendrocytes and astrocytes) are also present in the cell mixture. Most of the remaining genes showed no tissue enrichment, aside from JUP (enriched in the oesophagus and skin), TKT (bone marrow) and ACSS2/EEF1A2 (skeletal muscle). Of the brain-enriched or enhanced genes, INA, MAPT, NEFL, NSF, SNCA, SYN1, SYN2, SYT1, CAMK2B, CRMP1, DYNC1I1, RAB6B, L1CAM, MAP4K4, PHF24, TUBB2A and ATP1A3 were predominantly neuronal in expression, whereas LANCL1, MAP4K4, RAB33A, TUBB4A and SPOCK1 showed highest expression in oligodendrocytes and NCAN, GPM6B, PCDHGC3 and SLC1A3 in astrocytes. Of the remaining 24 genes which were not brain enriched, ACSL4, ATP6V1B2, EEF1A2, PCYOX1, MAPK1, PIP4K2B and YWHA showed highest expression in neurons, with the remainder being most highly expressed in oligodendrocytes (SUN2, FSCN1, JUP, ACSS2, FSCN1), astrocytes (MSN, G3BP1, FYN), microglia (GPX1), or showing no notable association with cell-type expression (MSMO1, PTBP1, TXNDC5, FABP5, MYL12B, PPP2R1A, TKT, VPS4A).

Several of these genes are involved in human diseases, including JUP (Cardiomyopathy), MSN (Immunodeficiency 50), SNCA (Parkinson’s disease, Lewy Body Disease), MAPT (Frontotemporal Dementia), NEFL (Charcot-Marie-Tooth) SYN1 (Epilepsy, X-linked), TUBB2A (cortical dysplasia), TUBB4A (torsion dystonia, hypomyelinating leukodystrophy), ATP1A3 (dystonia, epileptic encephalopathy, hemiplegia of childhood, CAPOS syndrome), and GPX1 (hemolytic anemia). SLC1A3 is discussed in the previous section on genes showing expression changes. It was the only gene showing significant changes in expression at two time points in addition to proteomic change. DYNC1I1 has also been associated with Lewy body pathology, where it is found in Lewy bodies in autopsied human brain^50^. A key molecular feature of neurodegeneration is deficits in microtubule‐based cargo transport and DYNC1I1 is involved in excitotoxicity induced axonal degeneration^51^.

We examined differential expression of these genes in human, post-mortem AD and control brain and in a series of tau and amyloid models of AD/tauopathies (Supplementary File 5). Although a number of genes showed significant changes in expression in one or more brain regions, most of these were small in magnitude. MSN (log2FC 0.50, human temporal cortex) was upregulated in several APP and MAPT models of AD, including Tg4510 at 4.5 and 6 months and TauD35_aged_HC. NEFL (log2FC − 0.43), SNCA (log2FC − 0.28) and NSF (log2FC − 0.34) were both downregulated in human temporal cortex and in tau models (log2FC − 0.29 Tg4510_6_FB_fem, log2FC − 0.28 TauD35_aged_HC and log2FC − 0.38 MAPT_P301L_12_SC_fem respectively). In the present study however, NEFL, SNCA and NSF were all upregulated at the transcript level, although downregulated at the protein level.

The most common theme among the full set of genes was involvement in the cytoskeleton. These genes included two members of the CRMP family (microtubule-associated major phosphoproteins in the developing nervous system) CRMP1 and DPYSL2 (aka CRMP2) which are prominent in nervous system development, including axon guidance, synapse maturation, cell migration, and adult brain function, DYNC1I1 (microtubule motor activity), FSCN1 (actin filament binding), INA (structural constituent of postsynaptic actin cytoskeleton), JUP (cytoskeletal protein-membrane anchor activity), TUBB2A, TUBB4A (both major components of microtubules), all showing transcript and protein increase, MSN (actin binding), all showing decreases in protein and transcript), MAPT (microtubule binding), NEFL (microtubule cytoskeleton organization), SNCA (actin binding), STMN1 (regulation of microtubule polymerization or depolymerization) and SYN1 (actin binding), increase in transcription but decrease in protein). This is interesting because MAPT (tau), the seed protein used in these experiments is known to promote microtubule assembly and stability. Most of these microtubule-related genes show upregulation at the transcript and protein level—the mechanisms of this are not clear but may relate directly to the effects of tau seeding on the microtubule system. The downregulation of MAPT, NEFL and SNCA after tau seeding might represent sequestration of these protein by aggregating tau or loss from the cells.

Two interacting genes identified as altered at both the transcript and protein level are INA (internexin A) and NEFL (neurofilament light). These two proteins, together with neurofilament H (NEFH) form neurofilaments, intermediate filaments that provide structural support for the asymmetric geometries of neurons and the radial expansion of myelinated axons for effective nerve conduction velocity^52^. INA is a Class-IV neuronal intermediate filament that can self-assemble into an independent structural network or cooperate with NEFL to form a filamentous backbone with NEFM and NEFH cross-bridges^52^. Experimental evidence suggests that INA interacts with both APP and SNCA (Uniprot; IntAct). NEFL is also an established biomarker of early neuronal injury and axonal degeneration that has been shown to be elevated in preclinical AD^53^. For example, pre-symptomatic PSEN1 mutation carriers had higher plasma NfL levels than non-carriers and higher NfL levels were associated with greater regional tau burden and worse cognition, but not amyloid β load^54^. INA showed an increase in transcript but both an increase and decrease in protein, for different peptides, whereas NEFL shows an increase in transcript and a decrease in protein. In the case of INA, these changes may represent isoform switching, although the INA gene has only three exons and does not show obvious evidence of splicing (GTEx). It is plausible that the presence of tau seed in these cells causes gene expression changes through interference in neurofilament formation.

Mutations in neurofilament genes cause several neuroaxonal disorders characterized by disrupted subunit assembly and neurofilament aggregation. These include Charcot–Marie–Tooth disease CMT2E, CMT1F and CMTDIG (NEFL mutations; OMIM) and axonal Charcot–Marie–Tooth disease type 2CC (CMT2CC; NEFH mutations; OMIM). Although INA is not associated with human diseases, accumulation of α-internexin is a neuropathological hallmark of NF protein aggregates in neurofilament inclusion body disease (NIBD), a form of frontotemporal dementia^55^.

There were several other themes associated with the transcript and expression alterations, including phospholipid metabolism (PIP4K2B, ANXA7, SYT1, SNCA), lipid metabolism (ASCL4, FABP5, EEF1A2, ACSS2), autophagy and vesicle transport (ANXA7, EEF1A2, PIP4K2B, ATP6V1B2, VPS4A, SNCA, MAPT, RAB33A), lysosomal biology (VPS4A), ion transport (PHF24, ATP1A3) and apoptosis (SNCA, TXNDC5, PPP2R1A, FYN, GPX1). EEF1A2 is the gene for Developmental and epileptic encephalopathy 33 and Mental retardation, autosomal dominant 38. ATP6V1B2 is associated with Congenital deafness associated with onychodystrophy. Defects in L1CAM are associated with L1 syndrome, which encompasses a wide phenotypic spectrum from severe hydrocephalus and prenatal death (HSAS) to a milder phenotype (MASA), even occur within the same family. (OMIM).

Several of these proteins also interact directly with tau, including FYN and α-synuclein. α-Synuclein and tau aggregates are the neuropathological hallmarks of Parkinson's disease (PD) and AD, respectively. The direct binding of α-synuclein and tau may act synergistically with α‐synuclein pathology to confer a worse prognosis, perhaps through forming hybrid oligomers^56^. Similarly, α-synuclein has been shown to modulate tau spreading in mouse brains^57^. Ppp2r1a interacts with Gna12, which promotes dephosphorylation of tau^58^.

PTBP1 plays a role in pre-mRNA splicing and regulation of alternative splicing events and is thought to represses the splicing of MAPT/Tau cassette exon 10^59^. Alternative splicing of MAPT cassette exon 10 produces tau isoforms with four (4R) or three (3R) microtubule-binding repeat domains in an approximately equal physiological ratio: deviations from this ratio in human neurons can lead to human neurodegenerative disorders, such as frontotemporal dementia with Parkinsonism linked to chromosome 17 (FTDP-17) and other disorders in which this ratio is altered^60,61^.

PIP4K2B is also one of the top 12 genes co-expressed with MAPT in human brain (Cell Type Expression Correlates^62^). The function of PIP4K2B is not well understood; it is a type 2 phosphatidylinositol-5-phosphate 4-kinase, generating second messengers, diacylglycerol and inositol-1,4,5-trisphosphate for phosphatidylinositol-4,5-bisphosphate (PI-4,5-P2) which is critical for synaptic vesicle docking and fusion. Pip4k2b in the mouse brain is expressed developmentally in neuronal populations, especially hippocampus and cortex, where it is co-localised with the marker NeuN. It is present in axon terminals and dendritic spines adjacent to the synaptic membrane, which support a potential role in synaptic transmission^63^.

Two of the genes identified in this paper have been associated with inflammatory processes in AD. MSN (moesin) is part of the ezrin-radixin-moesin (ERM) protein family that tether actin filaments to the plasma membrane when activated by phosphorylation and regulates cell shape, membrane transport, and signal transduction. Flow-cytometric microglial sorting coupled with quantitative proteomics identified moesin as a highly-abundant microglial protein with relevance to AD^64^. Moesin is expressed in plaque-associated microglia and nearly exclusively found in microglia that surround Aβ plaques in 5xFAD mouse brains^64^. In the present study it was decreased at both the transcript and protein level, which is opposite to the direction of effect seen in the 5xFAD mouse and human AD brain. It causes Immunodeficiency 50 (IMD50; OMIM) and has been identified as a potential therapeutic target and biomarker in Alzheimer’s disease^64^.

ANXA7 is also associated with neuroinflammation—it is a calcium/phospholipid-binding protein which promotes membrane fusion and is involved in exocytosis. It plays a role in autophagy, and also acts as an ion channel. ANXA7 plays a pivotal part in the process of Parkin-dependent mitophagy via interacting with BASP^65^. It is a biomarker of the inflammatory microglia phenotype in AD damaged tissues and is known to be involved in the inflammation processes and in microtubule network assembly rate^66^.

Taken together, we have developed a cellular model of tauopathy that recapitulates features of AD both at the transcript and protein level over time, providing a suitable platform with which to perform high-throughput perturbation screens for target or hit compound identification and/or validation.

## Supplementary Information

Supplementary Legends.

Supplementary Information 1.

Supplementary Information 2.

Supplementary Information 3.

Supplementary Information 4.

Supplementary Information 5.

Supplementary Figures.
