# Supplementary material for: A human tau seeded neuronal cell model recapitulates molecular responses associated with Alzheimer’s disease
Source: Sci Rep. 2022 Feb 17;12:2673. doi: 10.1038/s41598-022-06411-4 (PMC8854741; doi:10.1038/s41598-022-06411-4)
Supplement: Supplementary file 1 — Supplementary Legends. [file 41598_2022_6411_MOESM1_ESM.docx]

Files for Submission

Supplementary File 1 = Merged Transcriptomics and Proteomics Dataset

Supplementary File 2 = Data to generate Figure 2 heat map

Supplementary File 3 = Functional Enrichment Analysis Output

Supplementary File 4 = Spearman’s Correlation Analysis Output

Supplementary File 5 = Annotation File of 26 Genes with expression changes
