# Supplementary material for: A human tau seeded neuronal cell model recapitulates molecular responses associated with Alzheimer’s disease
Source: Sci Rep. 2022 Feb 17;12:2673. doi: 10.1038/s41598-022-06411-4 (PMC8854741; doi:10.1038/s41598-022-06411-4)

Supplementary Information

Supplementary figure S1. qRT-PCR to validate RNA sequencing analysis. A set of genes whose expression was either continually increased (a) or decreased (b) by RNA-Seq were chosen for qRT-PCR validation. Panel (c) and (d) show qRT-PCR results of the 8 genes and data were plotted for each time point as Log2 of seeded condition expression / unseeded control.

Supplementary figure S2. Gene expression heatmap of 1075 significantly differentially expressed genes in the control and seeded groups at DIV3, DIV7 and DIV14.

Supplementary figure 1.

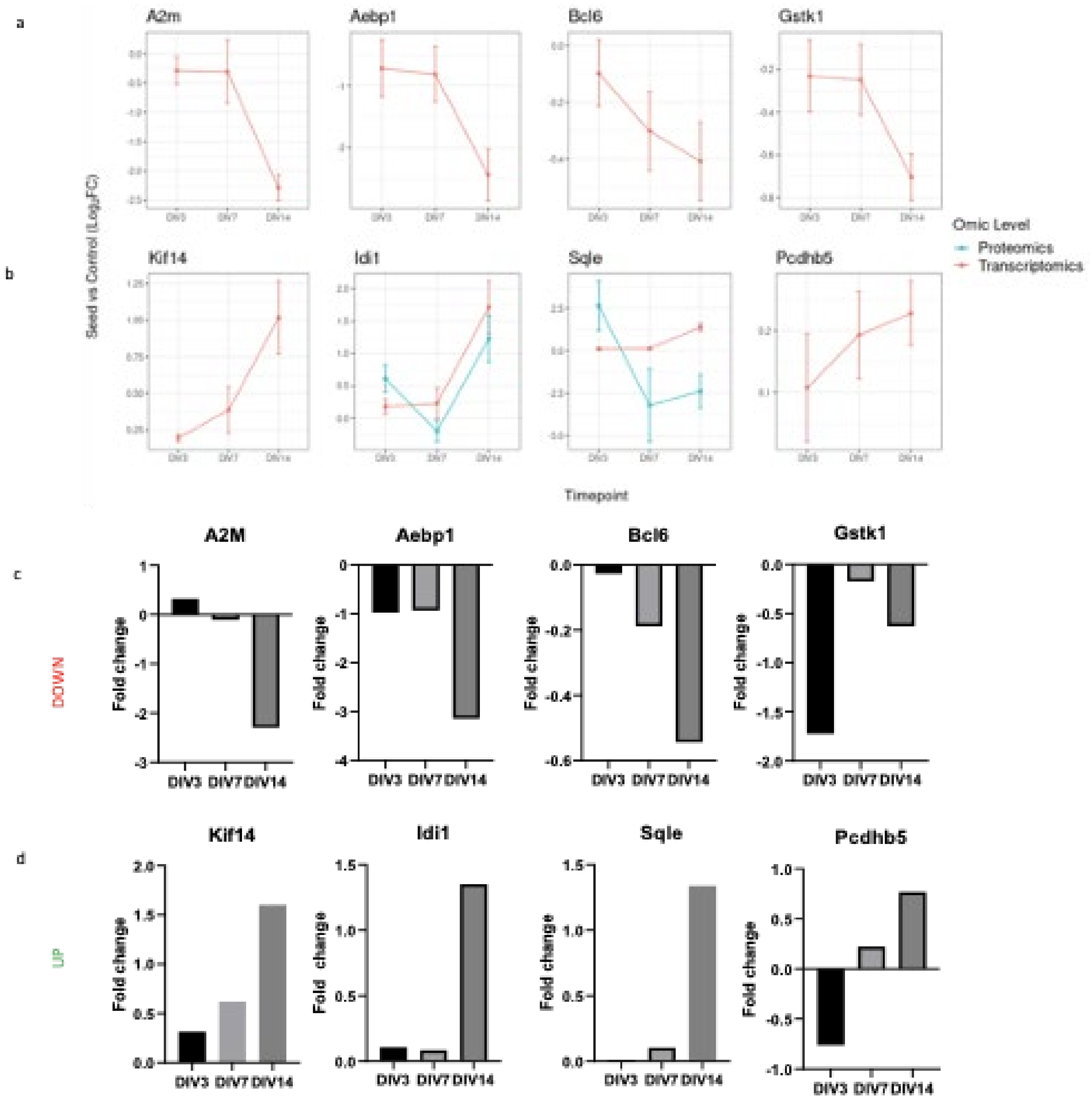

Supplementary figure 2.

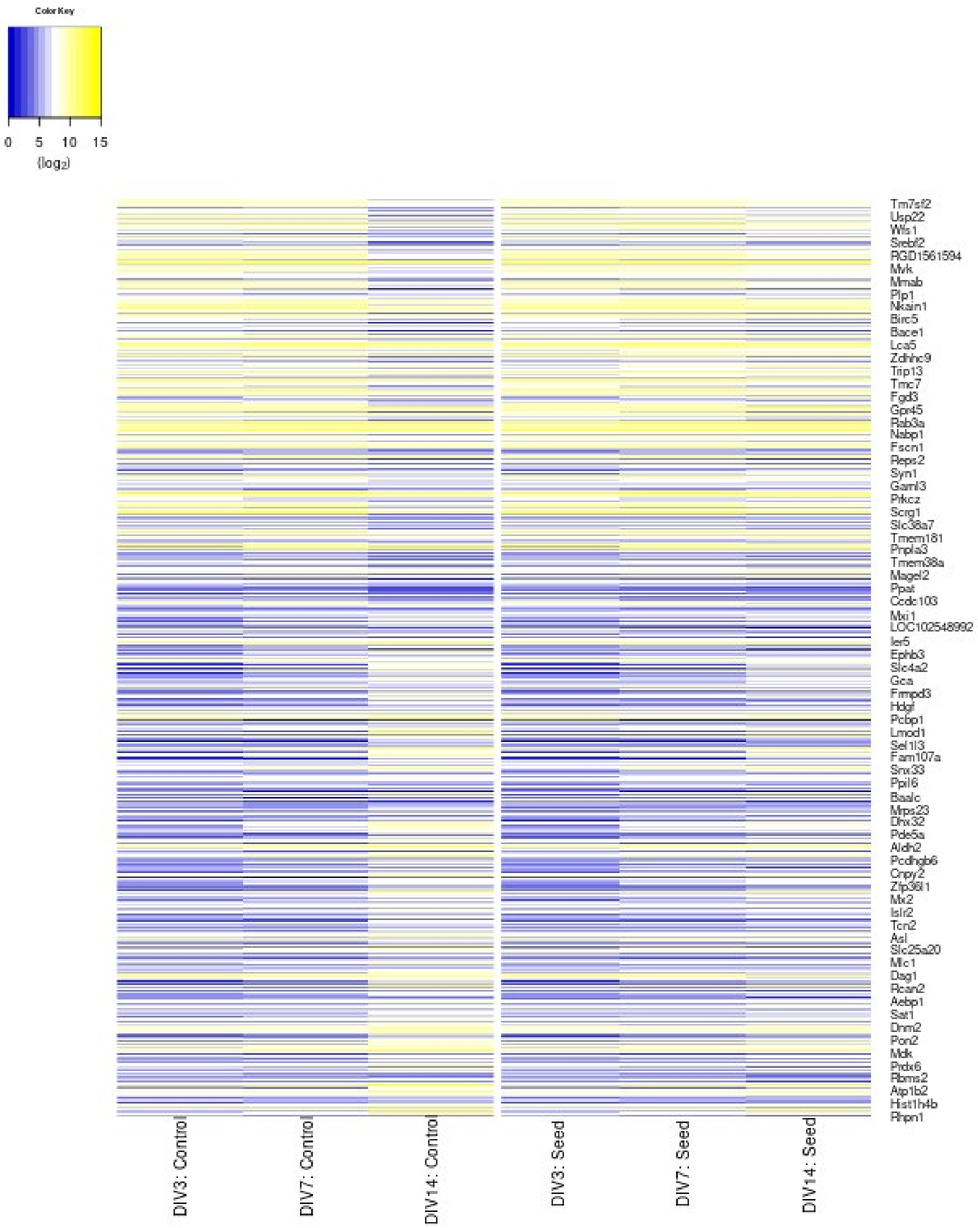

Supplement: Supplementary file 7 — Supplementary Figures. [file 41598_2022_6411_MOESM7_ESM.pdf]
